# Prevalence of autism spectrum disorder symptoms in a paediatric neurology clinic at a tertiary hospital in Uganda

**DOI:** 10.4102/sajpsychiatry.v27i0.1548

**Published:** 2021-01-28

**Authors:** Anita Arinda, Noeline Nakasujja, Raymond Odokonyero

**Affiliations:** 1 Department of Psychiatry, School of Medicine, Makerere University College of Health Sciences, Kampala, Uganda

**Keywords:** prevalence, associated factors, autism spectrum disorder, neurological disorders, Uganda

## Abstract

**Background:**

Children with neurological disorders are more likely to present with autism spectrum disorder (ASD) symptoms and get an ASD diagnosis. Despite the large burden of childhood neurological disorders in Uganda, there is limited information on ASD amongst children with neurological disorders in Uganda.

**Aim:**

The aim of this study was to determine the prevalence and factors associated with ASD symptoms amongst children attending the paediatric neurology clinic.

**Setting:**

The study was conducted at the paediatric neurology clinic of Mulago National Referral Hospital in Uganda.

**Methods:**

This was a cross-sectional study of 318 children aged 2–9 years. After obtaining consent, a socio-demographic questionnaire and the Social Communication Questionnaire were administered to the caregivers of the children. Additional questions were administered to assess the prenatal, birth and postnatal characteristics of the children. Sample characteristics were described using frequencies and means. Bivariate analysis was carried out using chi-square test and Fisher’s exact test. Multiple logistic regression models were used to assess which factors were independently associated with ASD symptoms.

**Results:**

The mean age of the children was 5 years and 58.2% were males. The prevalence of significant ASD symptoms was found to be 45%. Factors negatively associated with significant ASD symptoms were female sex (odds ratio [OR] 0.48 [95% CI 0.24, 0.98]) and ability to speak (OR 0.09 [95% CI 0.04, 0.2]). The history of delayed developmental milestones was positively associated with significant ASD symptoms (OR 3.3 [95% CI 1.59, 6.84]).

**Conclusion:**

The prevalence of ASD symptoms is high in children with neurological disorders. Children, especially those with delayed developmental milestones, should routinely be screened for ASD.

## Introduction

Autism spectrum disorder (ASD) is a neurodevelopmental disorder characterised by persistent deficits in social communication and social interaction and restricted and repetitive behaviours, interests and activities.^1^

These deficits occur in multiple contexts and are characterised by deficits in socio-emotional reciprocity, verbal and non-verbal communication used for social interaction and difficulty in developing and maintaining relationships.^2^ These features vary depending on age, culture and intellectual ability. Verbal communication deficits range from speech delay, monotonous speech, echolalia and poor comprehension of speech to a complete lack of spoken language. Non-verbal communication deficits include poor eye contact, difficulty in use and understanding of facial expression or gestures. An early feature of social communication deficit in ASD is impaired joint attention that manifests as lack of pointing, showing or bringing objects to share interests with others or failure to follow someone’s pointing or eye gaze.^3^ Deficits in socio-emotional reciprocity, which is the ability to engage with others and share feelings, include little or no initiation of conversation or other forms of interaction with others and decreased or absent imitation of others. Deficits in developing, maintaining and understanding relationships may present as absent, reduced or atypical social interest, manifested by rejection of others, passivity or inappropriate approaches that seem aggressive or disruptive. These difficulties are particularly evident in young children, in whom there is often a lack of shared social play, imagination and insistence on playing by very fixed rules. Children with ASD also have varying presentation of restricted and repetitive behaviours. Repetitive behaviour may include stereotypical motor behaviour such as hand flapping, repetitive use of objects like lining up toys, or repetitive speech (echolalia), whilst restricted behaviour is characterised by insistence on sameness or highly fixated interests.

According to a mini review by Suresh and colleagues, the global mean prevalence of ASD was estimated at 26.7 per 10 000 population.^4^ However, children with neurological disorders are reported to have increased chances of receiving an ASD diagnosis and higher rate of problems associated with ASD compared with those without neurological disorders.^5^ In a study by Ryland and colleagues amongst 11–13-year-old children, it was found that those with neurological disorders had significantly higher scores on a screening tool for ASD symptoms compared with those without neurological disorders.^6^ The findings of this study also showed that 14.1% of the children with neurological disorders had significant ASD symptoms. In a study amongst children with hydrocephalus, significant ASD symptoms were reported in 13% of children aged 5–12 years.^7^ The ASD symptoms in these children were significantly associated with having another neurological disorder, for example, cerebral palsy and epilepsy. Amongst children with epilepsy, the prevalence of significant ASD symptoms as determined by various screening tools has been estimated to range between 26% and 32%.^8,9^ A study amongst children with cerebral palsy also reported a high prevalence of ASD symptoms of 19% and these children scored higher on the Autism Spectrum Screening Questionnaire compared with their counterparts without cerebral palsy.^10^

Various factors have been postulated to be associated with ASD amongst children. Perinatal factors have been studied extensively and those found to be associated with ASD, include gestational diabetes, maternal infections and drug use during pregnancy, caesarean delivery, preterm delivery, post-term delivery, low Apgar score and small for gestational age.^11^ Amongst the socio-demographic factors, high socio-economic status and male sex have been associated with ASD. Some specific factors that are significantly associated with ASD symptoms in children with neurological disorders include family history of mental and neurological disorders,^12^ the presence of comorbid psychiatric disorders like attention deficit hyperactivity disorder^10^ and intellectual disability.^13^

Neurological disorders in children represent a significant proportion of global burden of disease because they are a major cause of morbidity, disability, mortality and poor quality of life.^14^ Most of this burden occurs in resource-limited settings of Asia and Africa. In Uganda, there is a considerable burden of neurological disorders in children with epilepsy and motor disorders, cerebral palsy being the most common.^15^ The prevalence of epilepsy in children in a recent population study in Uganda was found to be 10.3 per 1000 children,^16^ whilst that of cerebral palsy is estimated to be 2.9 per 1000 children.^17^ Despite the presence of literature suggesting comorbidity of ASD and neurological disorders, there is limited information on ASD amongst children with neurological concerns in Uganda. This descriptive cross-sectional study was designed to determine the prevalence of ASD symptoms amongst children attending the paediatric neurology clinic in Mulago Hospital and the factors associated with ASD symptoms in these children. In this study, we defined the prevalence of those children attending the neurology clinic over the 3 months of recruitment that had significant ASD symptoms.

## Methods

### Study design and setting

This was a cross-sectional study conducted in the paediatric neurology clinic of Mulago National Referral Hospital, which is one of the two national referral hospitals in Uganda. It is located in Kampala district, the capital city of Uganda, about 4 kilometres (km) from the city centre. It is the largest hospital in Uganda, with a bed capacity of 1500. The hospital provides both inpatient and outpatient services for patients from all over the country and training of students of all cadres. The paediatric neurology clinic is a specialised outpatient clinic, which receives children with neurologic disorders from all over the country. It runs twice a week and about 50 children are attended to on clinic days. The conditions managed in this clinic include epilepsy, cerebral palsy and muscular dystrophy. It is also in this clinic that a number of neurodevelopmental disorders including ASD are usually seen before they are sent to the child and adolescent psychiatry clinic for further assessment and management.

### Eligibility criteria

Parents or caregivers of children aged 2–9 years were approached to participate in the study. Those who consented to participation were included in the study. The age of 2 years was considered as the lower limit for this study because at this age parents or caregivers started noticing symptoms that are characteristic of ASD. The age of 9 years was considered as the upper limit so as to limit this study to the age group below adolescence (the World Health Organization defines adolescence as the age group of 10–17 years).

Children who were too sick and needed urgent medical attention and those whose caregivers lived with them for less than 6 months and as such had insufficient information about the behaviour of the child were excluded from the study.

### Sample size calculation

Sample size was calculated based on Leslie Kish’s (1965) formula. With an expected proportion of 29.3% of children having ASD (Mpaka et al., 2016) and a standard error of ± 5%, the sample size was estimated at 318.

### Study procedure

The study participants were enrolled from the paediatric neurology clinic of Mulago Hospital between November 2018 and January 2019. Every child aged 2–9 years was considered as the potential participant for the study.

On a clinic day, the principal investigator and two research assistants (who were mental health nurses) approached the potential participants and their caregivers to assess for eligibility and interest in the research project. They explained the purpose of the study and procedures and obtained informed consent from the parents/caregivers. The research questionnaires took 45 min to complete. Those who were found to have significant ASD symptoms were directed to the child and adolescent mental health clinic to receive further assistance.

### Study measures and instruments

#### Autism spectrum disorder symptoms

Autism spectrum disorder symptoms are impairments in social communication, social interaction and restricted, repetitive patterns of behaviour as seen in children with ASD. These were measured using the Social Communication Questionnaire (SCQ).^18^ This 40-item questionnaire was researcher-administered and used to obtain information about the child’s behaviour from the caregiver. Of the 40 items, 27 items address deficits in social communication and interaction, whilst eight questions are focused on restricted, repetitive patterns of behaviour, interests or activities. Each item is scored ‘yes/no’, with a score of 1 or 0 to indicate the presence or absence of a given abnormal behaviour. The items are summed up to give a total score of 0–39 for verbal children and 0–33 for non-verbal children.

The SCQ is based on the Autism Diagnostic Interview-Revised,^19^ a semi-structured interview tool used as a gold standard for the diagnosis of ASD in individuals with a mental age of 18 months and above. The SCQ, initially validated amongst individuals of ages 4–40 years, was found to have a sensitivity of 85% and specificity of 75%.^18^ Subsequent validation studies that were conducted in only children above 4 years found sensitivity scores ranging from 88% to 97.4% and specificity values from 62% to 96.5%.^20,21,22^ This screening tool has also been used and validated in African settings. A validation study in Mali amongst individuals 4–20 years of age reported a sensitivity of 71% and a specificity of 72%,^23^ whilst a South African study amongst children of 2.5–14 years found a sensitivity of 77% and specificity of 100% if a cut-off of 15 was used.^24^ Many studies have been conducted to validate the SCQ in younger children. These studies conducted in children below 4 years have found sensitivities ranging from 79.6% to 93% and specificities from 40% to 89%.^25,26,27,28^ A cut-off of 15 has been shown to have a fairly good specificity and sensitivity in distinguishing between children with ASD and those with other developmental disabilities.^29^ In this study, significant ASD symptoms were defined as a score of greater than 15 on the SCQ.

#### Socio-demographic characteristics

Socio-demographic characteristics of both the child and the parents were assessed using a socio-demographic questionnaire. Child demographic characteristics included age, sex, education level, primary caregiver and family history of mental illness.

#### Perinatal characteristics

The following additional questions were asked to assess the prenatal, birth and postnatal characteristics: maternal and paternal age at the conception of the child, history of gestational diabetes and hypertension, mode of delivery, preterm delivery, birth weight, breastfeeding history and history of illness in the neonatal period.

#### Patient’s medical records

The participant’s clinical records obtained included the neurological condition, history of delayed milestones and the presence or lack of speech.

### Data analysis

Data analysis was carried out with STATA version 14. Frequencies and percentages were computed for the categorical variables, whilst means and standard deviation were used to describe the continuous variables. Chi-square tests and Fisher’s exact test were used for bivariate analysis. Variables whose level of significance was 0.2 or less at bivariate analysis were included in the multiple logistic regression model for multivariate analysis.

A *p*-value of less than or equal to 0.05 was considered to be statistically significant.

### Ethical consideration

This study was approved by Makerere University School of Medicine Research and Ethics Committee (#REC REF 2018-110). The caregivers of all the children gave written informed consent and children of 8–9 years gave written assent to participate in the study.

## Results

### Socio-demographic characteristics

The sample included 318 children with neurological disorders. Of the participants, 185 (58.2%) were males, with a female to male ratio of 1:1.4. The participants’ age ranged from 2 to 9 years, with a mean age of 5.0 years. The rest of the participant characteristics are represented in Table 1.

**TABLE 1:** Socio-demographic and clinical characteristics of participants.

Variable	Frequency (*N* = 318)	Percentage
**Sex of the child**		
Male	185	58.2
Female	133	41.8
**Primary caregiver**		
Both parents	229	72.0
Mother	61	19.2
Father	13	4.1
Other†	15	4.7
**School attendance**		
Yes	125	39.3
No	193	60.7
**Family history of mental illness**		
Yes	43	13.5
No	275	86.5
**Neurologic condition**		
Epilepsy	182	57.2
Cerebral palsy	110	34.6
Others‡	26	8.2
**Ability to speak**		
Yes	188	59.1
No	130	40.9
**Delayed developmental milestones**		
Yes	182	57.2
No	136	42.8

† , Aunt, grandmother, foster parents.

‡ , Erb’s palsy, Global Developmental Disorder, Myasthenia Gravis, Sickle cell disease, Stroke.

### Clinical characteristics

The children in this clinic presented with a number of neurological conditions, with the most common being epilepsy (57.2%) and cerebral palsy (34.6%). The rest of the disorders are represented in Table 1. More than half of the participants (182; 57.2%) had a history of delayed developmental milestones (sitting, standing, walking and talking).

### Prenatal, birth and postnatal characteristics of the study participants

The majority of respondents reported no history of gestational diabetes (99.4%) or gestational hypertension (90.9%). The most common mode of delivery was normal delivery (77%), followed by caesarean delivery (19.2%). The rest of the perinatal factors are presented in Table 2.

**TABLE 2:** Prenatal, birth and postnatal characteristics of the participants.

Variable	Frequency (*N* = 318)	Percentage
**Parental characteristics**		
**Maternal age at conception**		
< 40 years	314	98.7
≥ 40 years	4	1.3
**Paternal age at conception**		
< 40 years	278	88.3
≥ 40 years	37	11.7
**History of gestational hypertension**		
Yes	29	9.1
No	289	90.9
**History of gestational diabetes**		
Yes	2	0.6
No	316	99.4
**Preterm birth**		
Yes	38	11.9
No	280	88.1
**Mode of delivery**		
Normal delivery	245	77.0
Instrumental delivery	12	3.8
Caesarean delivery	61	19.2
**Child characteristics**		
**Birth weight**		
< 2.5 kg	31	10.2
2.5 kg – 4.0 kg	264	87.1
> 4.0 kg	23	7.2
**Illness in neonatal period**		
Yes	144	45.3
No	174	54.7
**Duration of breastfeeding**		
Never	7	2.2
< 6 months of age	20	6.3
≥ 6 months of age	291	91.5

### Prevalence and factors associated with significant autism spectrum disorder symptoms

This study found that 143 participants (45%) had significant ASD symptoms, that is, screened positive on the SCQ (above the cut-off of 15).

Table 3 illustrates the bivariate analyses of socio-demographic characteristics and clinical characteristics. The factors that were significantly associated with significant ASD symptoms on bivariate analysis included not attending school, lack of speech and delayed developmental milestones.

**TABLE 3:** Bivariate analysis of socio-demographic and clinic characteristics of the study participants.

Variable	SCQ score ≤ 15† *N* = 175		SCQ score > 15‡ *N* = 143		*p*
	*n*	%	*n*	%	
**Sex of child**					
Male	96	54.9	89	62.2	0.185
Female	79	45.1	54	37.8	
**Primary caregiver**					
Both parents	133	76.0	96	67.1	0.212
Mother	8	4.5	5	3.5	
Father	28	16.0	33	23.1	
Other§	6	3.5	9	6.3	
**School attendance**					
Yes	110	63.5	15	10.5	< 0.001
No	65	36.5	128	89.5	
**Family history of mental illness**					
Yes	25	14.3	18	12.6	0.660
No	150	85.7	125	87.4	
**Neurological condition**					
Cerebral palsy	16	9.1	94	65.7	< 0.001
Epilepsy	144	82.3	38	26.6	
Other¶	15	8.6	11	7.7	
**Ability to speak**					
Yes	157	89.7	31	78.3	< 0.001
No	18	10.3	112	78.3	
**History of delayed milestones**					
Yes	65	37.1	117	81.8	< 0.001
No	110	62.9	26	18.2	

SCQ, Social Communication Questionnaire.

† , Non-significant ASD symptoms.

‡ , Significant ASD symptoms.

§ , Aunt, grandmother, foster parents.

¶ , Erb’s palsy, Global Developmental Disorder, Myasthenia Gravis, Sickle cell disease, stroke.

Table 4 illustrates the bivariate analyses of the perinatal characteristics. The history of illness in the neonatal period was significantly associated with significant ASD symptoms.

**TABLE 4:** Logistic regression estimates of factors associated with screening positive for autism spectrum disorder.

Variable	Unadjusted OR		*p*	Adjusted OR		*p*
	OR	95% CI		OR	95% CI	
**Sex**						
Male	1	-	-	1	-	-
Female	0.74	0.47, 1.16	0.185	0.48	0.24, 0.98	0.044
**School attendance**						
Yes	1	-	-	1	-	-
No	0.07	0.04, 0.13	< 0.001	0.45	0.19, 1.07	0.07
**Neurological condition**						
Others†	1	-	-	1	-	-
Cerebral Palsy	8.01	3.13, 20.54	< 0.001	2.39	0.72, 7.94	0.155
Epilepsy	0.36	0.15, 0.85	0.019	0.64	0.21, 1.96	0.434
**Ability to speak**						
No	1	-	-	1	-	-
Yes	0.03	0.02, 0.06	< 0.001	0.09	0.04, 0.2	< 0.001
**History of delayed developmental milestones**						
No	1	-	-	1	-	-
Yes	7.62	4.51, 12.86	< 0.001	3.3	1.59, 6.84	0.001
**Neonatal illness**						
No	1	-	-	1	-	-
Yes	3.40	2.14, 5.40	< 0.001	1.46	0.72, 2.96	0.300
**Duration of breastfeeding**						
Never	1	-	-	1	-	-
< 6 months of age	0.6	0.09, 3.89	0.592	0.56	0.03, 11.23	0.708
≥ 6 months of age	0.31	0.06, 1.6	0.160	1.07	0.07, 15.81	0.959

OR, odds ratio.

† , Erb’s palsy, Global Developmental Disorder, Myasthenia Gravis, Sickle cell disease, stroke.

The results of the multivariate logistic regression analysis indicated that females were 52% less likely to screen positive for ASD (odds ratio [OR] 0.48 [95% CI 0.24, 0.98]). The children who had the ability to speak were 91% less likely to screen positive for ASD compared with those who were unable to speak (OR 0.09 [95% CI 0.04, 0.2]). Those with delayed milestones were 3.3 times more likely to screen positive for ASD than those without a history of delayed milestones (OR 3.3 [95% CI 1.59, 6.84]).

## Discussion

This study was designed to determine the prevalence and factors associated with ASD symptoms amongst children attending the paediatric neurology clinic of Mulago Hospital. The prevalence of significant ASD symptoms was estimated to be 45.0% in this population. Female sex and ability to speak were negatively associated with significant ASD symptoms, whilst history of delayed milestones was positively associated with significant ASD symptoms.

The estimate of prevalence is consistent with findings from a number of other studies that have shown ASD symptoms to be higher in children with neurological disorders compared with healthy peers or children with other chronic disorders.^6,8^ This high prevalence can be attributed to the nature of the setting from which the study population was obtained. This paediatric neurology clinic, which is situated at the national referral hospital, is a specialised clinic that provides medical care to children with some of the most severe neurological concerns that are referred from other health facilities. A number of factors may account for the increased prevalence of ASD symptoms in children with neurological disorders. An overlap between aetiological mechanisms and symptoms of the neurological disorders and ASD may contribute to the co-occurrence of neurological disorders and ASD symptoms.^30,31^ A number of children with neurological disorders also present with difficulties in cognitive functioning, especially intellectual disability, which may lead to decreased social functioning and deficient social relationships and may present with symptoms characteristic of ASD.^32^ In addition, the disabilities that commonly occur in neurological disorders also increase chances of children being rejected by others, thus affecting social relationships with their peers.^33^

The prevalence in this study, however, is higher than that shown in other studies that looked at ASD symptoms in neurological disorders. A study by Ryland and colleagues, which assessed for ASD symptoms amongst children with neurological disorders, reported a prevalence of 14.1%.^6^ Their prevalence could have been lower because they considered an older age group of 11–13 years. In addition, they considered only schoolgoing children that possibly ruled out more impaired children who usually fail to go to school. It is also worth noting that a different tool was used for assessment and hence produced different results. Other studies focusing on ASD symptoms, in particular neurological disorders including epilepsy and cerebral palsy, have also reported a lower prevalence compared with our study. Clarke and colleagues found a prevalence of 32% in children with epilepsy attending tertiary care,^34^ whilst Bjorgaas and colleagues found a prevalence of 20% in children with cerebral palsy.^10^ These studies, however, only considered schoolgoing children of ages different from those in our study (2–18 years and 11–13 years, respectively) and also ruled out intellectual disability. Some studies have shown that children with epilepsy and intellectual disability are at substantially increased risk of autism relative to those with epilepsy who are of normal intellectual abilities.^35^ It is, however, also important to note that this study was carried out in a lower resource setting compared with the given studies. In these settings, services for disability are scarce and thus it is the children with more severe disability that are brought to health facilities, whilst the milder cases do not enter the healthcare system. Therefore, this may explain why a large number of children in this clinic presented with ASD symptoms that may have complicated the already existing neurological condition and thus prompting caretakers to seek medical care.

### Factors associated with autism spectrum disorder symptoms

According to this study, being male was positively associated with screening positive for ASD symptoms. This is in line with study findings that indicate the rate of ASD to be significantly higher in males than in females, with a frequently stated male to female ratio of 4:1.^36,37,38^ The explanation for this difference is not clear but there are various theories to try and explain this. One of the explanations is the ‘empathising–systemising’ theory, which postulates that typically females exhibit more empathising (able to identify oneself with other’s mental state and to respond to these feelings and thoughts with an appropriate response) and less systemising (ability to analyse and build systems) compared with typical males.^39^ Females with ASD may also display superficial social skills, which may mask ASD symptomatology impacting the identification of the disorder.^40^ This has been termed as the ‘camouflage’ hypothesis. Compared with their male counterparts with ASD, it is increasingly recognised that females with ASD have a stronger ability to imitate socially acceptable behaviour, particularly the females with higher cognitive abilities.^40^ This capacity to camouflage social difficulties in social situations is considered to be one of the main features of the female phenotype of ASD.

Social imitation or camouflaging enables some level of social success and coping, which results in some females never receiving the diagnosis of ASD because they may not exhibit any observable functional impairment.^41^ An alternative biological explanation suggested is the notion of ‘female-specific protective factors’ (FPF), which suggests that females can withstand a larger etiological load than males before reaching diagnostic thresholds of ASD.^42^

There was also a significant association between inability to speak and significant ASD symptoms. The lack of expressive speech has been described as a common finding in African children with ASD.^43,44^ Lagunju and colleagues reported that more than 50% of children with ASDs in a neurology clinic in Nigeria were non-verbal.^45^ Bakare et al. suggested that selection of severe cases in presentation to medical care may be a potential contributor especially in the African context.^43^ Some validation studies have reported increased sensitivity of the SCQ amongst non-verbal children.^46^ Non-verbal children have also been shown to score higher on the SCQ despite the fact that they have no data for six items that are strictly applicable to verbal children.^47,48^ One explanation given for this finding is that non-verbal children may show more severe features of ASD compared with their verbal counterparts.^48^

This study also showed a significant association between delay in milestones and ASD symptoms. This has been noted before in another study that reported delays in motor milestones in children with ASD compared with normal children.^49,50^ The motor delays in early childhood may contribute to later developmental delays in verbal and gestural communication, a characteristic of ASD.^51^ They may also negatively affect balance, social appearance and motivation to engage in social activities involving gross motor behaviours, for example, playing ball games.^52^ Furthermore, motor delays can influence frequency of challenging behaviour, especially avoidant behaviour, such as tantrums, commonly reported in children with ASD.

Education attainment was not found to be correlated with screening positive for ASD in this study. Amongst the children who screened positive for ASD, the number of those who were not attending school was significantly higher than those who were attending the school. However, on multivariate analysis, this association became insignificant. This is not in line with studies that have reported that ASD negatively affects education attainment amongst affected individuals.^53^ The findings in this study may be explained by the fact that even the children that screened negative for ASD symptoms have other factors that may also affect school attendance in these children, for example, socio-economic status and age.

There was no association between perinatal factors and ASD in this study. There have been mixed findings about this association in various studies,^11^ with some studies reporting a positive association^54,55^ whilst others reporting no association.^56,57^ The only factor that has consistently shown an association is advanced paternal age. In our study, there were a small number of fathers above 40 years of age and this could have affected these results.

### Strengths and limitations

The main strength of this study was that it had a large sample size that increased the power of this study and the generalisability of the results. This study adds to the limited database of ASD within the African context.

This study had several limitations. Firstly, this is a cross-sectional study; thus, the temporal relationship between ASD and neurological disorders could not be established. Therefore, inferences about causation could not be made in this cross-sectional study because the exposure and outcome were measured at the same time. Secondly, there was no independent validation of ASD symptoms by direct assessment of the children by a qualified clinician. The SCQ cannot be used to make a diagnosis of ASD as this requires a more detailed description of actual behaviour, onset and development over time as well as effect on functioning.^18^ Parent or caregiver reports on a screening questionnaire with ‘yes’ or ‘no’ answers, therefore, cannot replace a clinical validation of diagnosis. Speech was also not assessed by a qualified specialist, but we mainly depended on parental reports and these findings may have not taken into account developmental age. Thirdly, there was potential for recall bias caused by requirement for respondents to provide information regarding experiences or events from the past. There are also limitations in the generalisability of these findings to other children with neurological conditions in other settings as this study was performed in a national referral hospital that usually receives the more severe cases.

### Implications and recommendations

The present findings highlight the need for awareness amongst clinicians about the likelihood that a number of children with neurological disorders in Uganda might be experiencing significant ASD-related challenges. It is, therefore, important that clinicians have access to and use screening instruments to enable them to identify these problems that may pose a great challenge to the affected children and their families. Emphasis can be placed on children who present with delays in developmental milestones.

Further research is recommended amongst those who screen positive using diagnostic tools such as the Autism Diagnostic Interview-Revised and the Autism Diagnostic Observation Schedule.

## Conclusion

This study found that almost half of the children attending a neurology clinic in a tertiary hospital in Uganda presented with significant ASD symptoms. The children with significant ASD symptoms were most likely males, had limited or no speech and history of developmental delays.
